# On the Ability of Bismuth to Couple Weakly Coordinating Anions

**DOI:** 10.1002/anie.6127002

**Published:** 2026-05-30

**Authors:** Lucas Mele, Reena Balhara, Dimitrios A. Pantazis, Josep Cornella

**Affiliations:** ^1^ Max‐Planck‐Institut Für Kohlenforschung Mülheim an der Ruhr Germany

**Keywords:** bismuth catalysis, driving force, relativistic effects, weakly coordinating anions

## Abstract

Similar to aryldiazonium salts, hypervalent bismuth species have been shown to promote coupling of weakly coordinating anions (WCAs) such as triflate and tetrafluoroborate. In this study, we identify the origin of the driving force for these transformations by quantifying scalar relativistic effects in high‑valent bismuth chemistry. All‑electron DFT comparisons between relativistic and non‑relativistic limits reveal pronounced stabilization of the Bi 6s orbital in the former framework. As a result, the structure and frontier‑orbital energetics of the Bi(V)/Bi(III) processes are heavily affected, thus having a pronounced effect on the reductive elimination. Specifically, relativistic contributions to Bi could be observed on different scales ranging from a simple monoatomic model to complex reaction profiles. These results rationalize the facile WCA coupling from catalytically relevant Bi(V) complexes and establish the basis of why Bi(III) is an exceptionally potent nucleofuge in bismuth‑mediated bond formation.

## Graphical abstract

Motivated by the growing number of catalytic processes based on high‐valent Bi, we investigated in silico the reductive elimination step responsible for the coupling of weakly coordinating anions. Relativistic contributions appeared to be decisive to the electronic description of the intermediates involved in this process. The thermodynamic driving force provided by Bi could be traced to scalar relativistic stabilization of its 6s orbital.

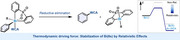

## 1 Introduction

**FIGURE 1:**
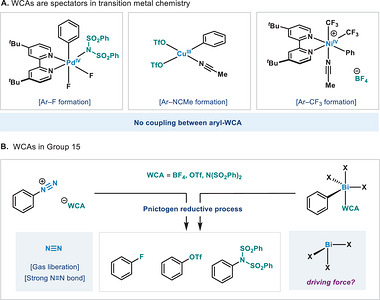
(A) Reductive elimination events from high‐valent transition metal complexes bearing spectating WCAs. (B) Couplings of WCAs in organo‐pnictogen compounds.

Weakly coordinating anions (WCAs) have long been utilized in the generation and stabilization of cationic metal complexes [1]. In stoichiometric organometallic redox chemistry and catalysis, common WCAs (tetrafluoroborate 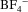, triflate TfO^−^, and bisphenylsulfonimidate NSI^−^) are routinely exploited to render complexes more electrophilic, permitting vacant sites for ligand coordination or substrate binding. Their electronic nature renders them intrinsically poor nucleophiles and generally remain inert spectating anions. It is for this reason that C─WCA bond‐forming events are normally not observed, even when the metal is in a high‐oxidation‐state, permitting the formation of other challenging bonds (Figure 1A). For example, the Sanford group reported that cationic Pd(IV) complexes bearing a bisphenylsulfonimidate anion resulted in the formation of the difficult C(sp^2^)─F rather than C(sp^2^)─NSI bond [2]. The challenging C(sp^2^)─CF_3_ reductive elimination from a high‐valent Ni(IV) complex also proceeds without side reactivity of the tetrafluoroborate anion [3]. Another remarkable example is the cationic Cu(III) center in Figure 1A. It has been shown to engage in reductive elimination with a neutral acetonitrile (MeCN) ligand instead of triflate, producing an unusual arylnitrilium cation [4], although triflate can occasionally react under specific conditions [5, 6]. In spite of their inertness in transition metal complexes, WCAs can engage in reactivity with certain main group compounds (Figure 1B) [7, 8]. The most notable example can be found in group 15, with the Balz–Schiemann reaction, where tetrafluoroborate (BF_4_^−^) serves as a fluoride source en route to fluoroarenes [9, 10]. This reaction is believed to proceed via a polar mechanism [11], in contrast to the radical pathway proposed for the Sandmeyer‐type reactions [12]. A less‐known and perhaps overlooked coupling of diazonium ions with WCAs is observed in the thermal decomposition of aryldiazonium triflates to afford aryl triflates [13]. In the same vein, aryldiazonium salts bearing NSI counterions also thermally decompose to the corresponding C(sp^2^)─N bond‐forming product [14]. In all these cases, the reaction is thermodynamically driven by the liberation of N_2_, facilitating a chemical step that would be otherwise challenging to achieve. The oxidation of the WCA is accompanied by the reduction of the N bonded to the aromatic ring of the diazonium group during these processes.

Descending group 15, aryl bismuthonium compounds exhibit parallel behavior to aryl diazonium salts. Seminal work by Wittig, Barton, Matano, Akiba, and Mukaiyama demonstrated that high‐valent bismuth species can undergo ligand–ligand coupling with diverse nucleophiles [15, 16, 17, 18], including fluorides [19] and triflates [20], with concomitant formation of Bi(III). Building on these precedents, our group recently developed catalytic variants of such couplings with WCAs, including triflate, nonaflate [21], tetrafluoroborate [22], and sulfonimidates [23] (Figure 1B). In all cases, the reductive elimination from isolated Bi(V) normally proceeds at 25°C, thus highlighting the strong tendency of Bi(III) to act as a leaving group. These transformations bear a striking analogy to diazonium‐mediated WCA activation, yet the origin of the thermodynamic driving forces is inherently different. As the heaviest stable pnictogen, bismuth's oxidation state preference (+III) is explained by the inert pair and relativistic effects, as studied by Pyykkö and others [24, 25, 26]. In the case of aryl diazonium salts, liberation of a gas and the high stabilization of the triple bond provide the driving force for the C─WCA bond formation [27]. On the other hand, the thermodynamic arguments for the driving force in the coupling of WCAs from Bi(V) are not trivial. In this report, we investigate the main contributors that lead to the observed reactivity, effectively enabling Bi(III) to act as a potent nucleofuge. This work aims to shed light on the influence of the stabilization of the 6s orbital as a crucial thermodynamic driving force in high‐valent bismuth processes. Through computational analysis, we investigate the energetics of various catalytically relevant reductive eliminations from Bi(V) and assess the contribution of relativistic effects to their feasibility.

## 2 Results and Discussion

To evaluate the role of relativistic effects, it is necessary to compare and contrast computed energetics from both scalar relativistic and non‐relativistic quantum chemical calculations [28, 29, 30, 31]. Here, we discuss energy differences obtained from single‐point all‐electron scalar relativistic and non‐relativistic density functional theory (DFT) calculations using a common reference set of geometries. These geometries were obtained from B3LYP [32, 33]/def2‐TZVP(‐f) [34] optimizations with the ECP60MDF small‐core effective core potential of Metz et al. on Bi [35], an approach that better isolates the purely electronic valence effects [35, 36, 37, 38]. We stress that numerical comparison of all‐electron relativistic and non‐relativistic results is meaningful only if both types of calculation are performed with exactly the same basis set. After extensive testing, we concluded that the all‐electron SARC basis sets [39, 40, 41] performed best among all other basis sets available in ORCA 6.1 [42]. Importantly, the highly flexible SARC basis sets for Bi [40] were also appropriate for non‐relativistic calculations, whereas most of the strongly contracted all‐electron relativistic basis sets, explicitly adapted to relativistic Hamiltonians, failed to yield convergence of the self‐consistent‐field (SCF) equations at the non‐relativistic limit. The comparison between relativistic and non‐relativistic calculations can be done with energetics obtained either on separately optimized molecular geometries or with the same geometries. We employed both approaches and found that they led to quantitatively identical conclusions regarding reaction energetics (complete data provided in the Supporting Information).

**FIGURE 2:**
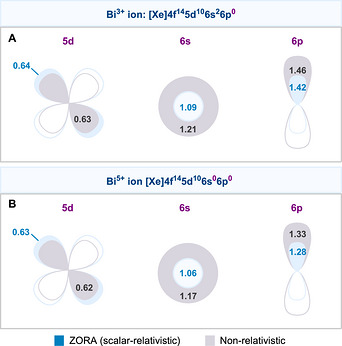
Radial expectation values (in Å) for Bi(III) (A) and Bi(V) (B) ions, obtained from ZORA and non‐relativistic Hartree–Fock calculations with the SARC‐ZORA‐TZVP basis set. Depictions represent visual schematic representations of the canonical orbitals and are not at scale. Light blue: relativistic; grey: non‐relativistic.

First, we evaluated the effects of relativity on the monoatomic Bi(III) (Figure 2A) and Bi(V) (Figure 2B) ions, at the level of theory used throughout this work. The term “relativistic effects” in molecular quantum chemistry encompasses several theoretically distinguishable contributions, formally defined through successive approximations to the full four‐component Dirac equation. Scalar relativistic (one‐electron) terms dominate the impact on molecular structure and reaction energetics [24, 43, 44, 45]. The primary scalar correction (mass‐velocity term) arises from the relativistic increase in the mass of inner‐shell electrons as their velocities approach the speed of light (*c*) for heavier elements. Relative to a fictitious non‐relativistic system, these effects lead primarily to contraction/stabilization of s orbitals and, to a lesser extent, p orbitals, with concomitant de‐shielding of d and f orbitals [46]. The results for the bare ions are consistent with these interpretations and with previous calculations [47], showing a pronounced contraction of the 6s orbital (≈ 0.12 Å), a moderate stabilization of 6p electrons (≈ 0.04 Å), and a negligible expansion of the core 5d shell, to a nearly identical extent for both ions. When considering the relativistic corrections, we note that although the radial expectation values of core d orbitals in the two ions are almost identical (≈ 0.63 Å), valence shells, specially p, are more diffuse for Bi(III) with a radius of 1.42 Å (Figure 2A) compared to 1.28 Å for Bi(V) (Figure 2B). Spin‐orbit coupling (SOC) was not included here as we focus on scalar relativistic effects. Nonetheless, thermodynamic and kinetic energies in high‐valent bismuth redox chemistry are largely dominated by changes in the 6s orbital, which is unaffected by SOC (vide infra).

**FIGURE 3:**
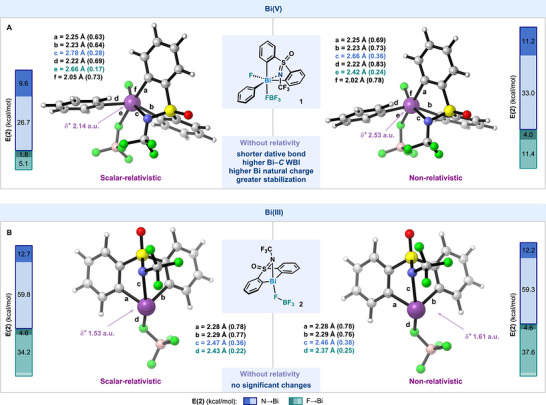
Structural analysis of complex **1** (A) and **2** (B) in both scalar‐relativistic and non‐relativistic scenarios. Bond length in Å, Wiberg bond indices (WBI) are indicated in parenthesis. Natural atomic charges on Bi are given in atomic units (a.u.). E(2) is the second‐order perturbation stabilization energy (kcal/mol) from NBO analysis for the Bi complexes **1** and **2** with donation from F to Bi in green and donation from N to Bi in blue.

We then investigated how these electronic effects influence the structures of key reaction intermediates in high‐valent bismuth redox catalysis [48, 49]. The system featuring a diaryl‐sulfone and ‐sulfoximine supporting the bismuth center has emerged as a privileged motif in catalysis [50, 51]. The model reaction we chose to explore was the catalytic platform for the fluorination of boronic esters [22]. Inspection of the intermediates relevant to reductive elimination (e.g., Bi(V) **1** and the Bi(III)‐based product **2**), at both the all‐electron relativistic and non‐relativistic limits, reveals major structural differences (Figure 3). Noteworthy, computed geometries at the relativistic level are consistent with the experimental obtained by single‐crystal x‐ray diffraction (see Figures S2 and S3) [22, 52]. As shown in Figure 3A, changes in the parent Bi(V) intermediates are more pronounced than in the corresponding Bi(III) complexes (Figure 3B). Whereas computed bond lengths in **2** remain nearly unchanged, dative bond lengths in the Bi(V) adduct **1** are elongated by up to *ca*. 0.2 Å relative to the non‐relativistic analogue. Moreover, the Wiberg bond indices for the Bi(V)─C bonds are higher in the non‐relativistic picture, suggesting greater stability of this compound in the fictitious non‐relativistic limit. Consistent with this picture, the Bi center in **1** displays a more pronounced cationic character in the non‐relativistic calculations, reflecting reduced core‐electron shielding. This trend is evident from the calculated natural atomic charges on Bi: 2.528 a.u. (non‐relativistic) versus 2.142 a.u. (relativistic). Consequently, the larger positive charge in the non‐relativistic case leads to stronger electrostatic interactions with the ionic BF_4_^−^ anion and the sulfoximine donor. Indeed, NBO second‐order perturbation theory analysis (E(2)) shows larger F→Bi and N→Bi stabilization energies in the non‐relativistic calculations, consistent with enhanced donor‐acceptor mixing (stronger overlap) between ligand‐ and Bi‐based orbitals. Specifically, electronic donation from the sulfoximine nitrogen to the bismuth center increases by 7.9 kcal/mol when relativistic considerations are obviated. Similarly, contribution from the *F*BF_3_ augments in the non‐relativistic limit with a stabilization energy difference of 8.5 kcal/mol. This stronger donor–acceptor interaction results in higher bond orders and shorter Bi─F and Bi─N bonds.

**FIGURE 4:**
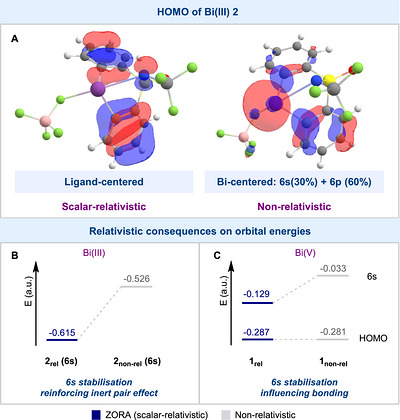
Electronic consequences of relativistic effects on **1** and **2**. (A) Changes in the HOMO of Bi(III) **2**. Orbital energies for complexes **1** (B) and **2** (C) using the B3LYP functional and the ZORA Hamiltonian with all‐electron SARC‐ZORA‐TZVP and ZORA‐def2‐TZVP basis sets.

The contrasted impact of relativistic effects on the Bi(III) and Bi(V) structures can be rationalized by the distinct chemical role of the Bi(6s) orbital in these two oxidation states. In the Bi(III) complex **2**, bonding is dominated by p‐interactions, and the 6s electrons largely constitute a spherical nonbonding lone pair (Figure 3B). We note that in the relativistic case the HOMO of the Bi(III) compound **2** is ligand‐centered and 6s orbital character is significantly stabilized, whereas in the non‐relativistic case the HOMO is Bi‐centered with substantial s–p mixing of *ca*. 30% s and 60% p (Figure 4A). This is clearly reflected in electronic terms, since the energy of Bi(III) **2** 6s orbital is lowered from −0.53 a.u. to −0.62 a.u., when considering relativistic contributions (Figure 4B). Nevertheless, even if the s character of the frontier orbitals differs between relativistic and non‐relativistic calculations, the inert‐pair nonbonding nature of the 6s orbital in Bi(III) results in minimal geometric differences between relativistic and non‐relativistic descriptions.

In contrast, the pronounced structural changes observed for **1** (Figure 3A) arise from the availability of the geometrically active 6s when engaged in bonding: upon inclusion of relativistic effects, the acceptor orbital with dominant Bi(6s) character is energetically stabilized by ca. 0.1 a.u. (Figure 4C). Therefore, scalar relativistic effects contract the Bi(6s) orbital, leading to lesser σ donation from highly electronegative ligands. Accordingly, the E(2) values and bond orders are lowered, which result in lengthening of the Bi─F and Bi─N bonds. Furthermore, the energy of the HOMO of Bi(V) complex **1** remains unchanged (≈ −0.28 a.u.) upon inclusion of relativistic effects (Figure 4C). Overall, since the vacant 6s orbital of Bi(V) is stabilized, these effects result in a smaller HOMO–LUMO gap.

This orbital‐energy rebalancing—relativistic contraction of Bi(III) filled 6s orbital and destabilization of the Bi(V) structure due to poor ligand donation—appears clearly in the computed energy. Specifically, the Bi(V)/Bi(III) computed energy difference is 40.1 kcal/mol at the relativistic limit, compared to 7.3 kcal/mol in the fictitious non‐relativistic model. This trend is consistent with the experimental requirement for strong oxidants, such as XeF_2_, *m*‐CPBA, SelectFluor, or *N*‐fluoropyridinium salts, to oxidize Bi(III) precursors [53].

**FIGURE 5:**
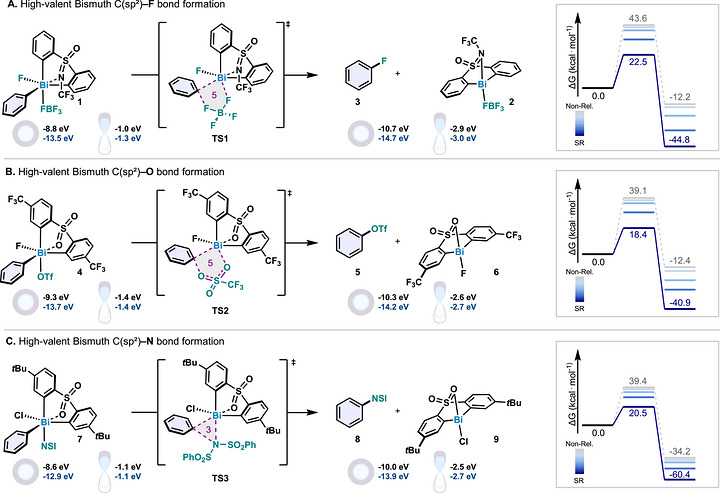
Reductive elimination from high valent bismuth. (A) Formation of aryl‐fluoride via a 5‐membered ring transition state. (B) Formation of aryl‐triflate via a 5‐membered ring transition state. (C) Formation of aryl‐NSI via a 3‐membered ring transition state. Scaled values of electronic energies (kcal/mol) with adjusted speed of light using the B3LYP functional and the ZORA Hamiltonian with all‐electron SARC‐ZORA‐TZVP and ZORA‐def2‐TZVP basis sets, with varying relativistic contribution. From light gray to dark blue: non‐relativistic, c/0.25, c/0.50, c/0.75, and ZORA. Calculated orbital energies (eV) for the valence s and p orbitals with the B3LYP functional and the ZORA Hamiltonian with all‐electron SARC‐ZORA‐TZVP and ZORA‐def2‐TZVP basis sets; non‐relativistic values in black, scalar‐relativistic (SR) values in blue.

Recognizing that relativistic effects are responsible for the large energy difference between Bi(III) and Bi(V), not only in monoatomic models but also in isolated complexes, we next investigated how this difference translates to reactivity, and thus why C─WCA bond formation becomes feasible. To this end, we probed the scalar relativistic contributions to three reported reductive eliminations: C(sp^2^)─F and C(sp^2^)─OTf bond formation proceeding via five‐membered‐ring ligand‐coupling [21, 22], and C(sp^2^)─NSI operating through a three‐membered ring process [23]. We employed three popular scalar relativistic Hamiltonians, the zero‐order regular approximation (ZORA) [54, 55], the second‐order Douglas–Kroll–Hess Hamiltonian (DKH2) [56, 57], and the scalar version of the exact 2‐component (X2C) Hamiltonian [58, 59]. The results document the drastic rebalancing of energetics in all reactions considered, with product formation becoming far less favorable in the non‐relativistic limit by ca. 30 kcal/mol for all reactions (Figure 5). All relativistic Hamiltonians considered (ZORA, DKH2, and X2C) yield practically equivalent results (see Table S1), suggesting that the conclusions do not depend numerically on the precise choice for the treatment of scalar relativity. It is noted that energetics from plain Effective Core Potentials‐based calculations are in line with the all‐electron scalar relativistic calculations, albeit do not coincide numerically. Calculations performed using fractions of the speed of light (*c*)—to effectively scale relativistic effects between the relativistic and non‐relativistic limits—show a smooth and regular progression as the fine structure constant *a* = 1/*c* is fractionally reduced (Figure 5). Finally, we note that explicit inclusion of SOC in two‐component X2C calculations conducted with Turbomole [60] and appropriate x2c‐TZVPall‐2c basis sets [61], confirmed that SOC has a minimal influence of ca. 1 kcal/mol on the overall thermodynamics of these reactions (see Table S2).

Together, these results trace the driving force for high‐valent bismuth‐mediated C─WCA bond formation to scalar relativistic effects. For example, in the triflation reaction, the transition state energy decreases from 39.1 kcal/mol in the absence of relativistic effect, to 28.9 kcal/mol with c/0.75 and 18.4 kcal/mol in the scalar relativistic model (Figure 5B). The overall thermodynamics of this transformation showed a similar behavior with ΔΔG decreasing from −12.4 to −40.9 kcal/mol upon inclusion of relativistic corrections. Consistent with this interpretation, natural atomic orbital (NAO) energies indicate substantial stabilization of the Bi 6s levels in scalar‐relativistic calculations relative to the non‐relativistic limit, whereas the Bi 6p levels are only weakly stabilized. This trend is in line with the work of Ferhat and Zaoui on bismuth diatomic molecules with B, Al, Ga, and In, where the calculated 6s levels without relativistic and with relativistic effects are −11.02 and −13.57 eV, respectively [62]. Scalar relativistic effects thus primarily stabilize valence s orbitals due to their strong nuclear penetration, while the influence on valence p orbitals is comparatively smaller. Beyond thermodynamics, we observe a regular increase in activation barriers upon decreasing relativistic effects, with transition‐state energies approximately doubling in the non‐relativistic limit (Figure 5). This trend also suggests that the success of high valent bismuth catalysis is inherently linked to the influence of relativity in bismuth.

## 3 Conclusion

In this report, we describe a straightforward computational protocol to quantify scalar‐relativistic contributions to reaction energetics. Applying this approach to high‐valent bismuth chemistry identifies scalar relativity as the principal origin of the driving force that enables the unusual coupling of WCAs. In particular, relativistic stabilization of the Bi 6s orbital dominates the Bi(III)/Bi(V) energetic gap, thereby providing a substantial thermodynamic bias toward reductive elimination. In this context, Bi(III) becomes an exceptionally effective leaving group that offsets the energetic penalty associated with WCA transfer. Overall, these results provide a mechanistic rationale for WCA activation by Bi(V) species and offer a general strategy to anticipate analogous thermodynamically driven transformations in the chemistry of bismuth and other heavy main‐group elements.

## Author Contributions

**Lucas Mele**: conceptualization, data curation, writing – original draft, and writing – review and editing. **Reena Balhara**: methodology, software, data curation, formal analysis, writing – original draft, and writing – review and editing. **Dimitrios A. Pantazis**: resources, funding acquisition, supervision, data curation, formal analysis, writing – original draft, writing – review and editing, and methodology. **Josep Cornella**: project administration, resources, funding acquisition, formal analysis, writing – review and editing, writing – original draft, conceptualization, and supervision.

## Conflicts of Interest

The authors declare no conflicts of interest.

## Supporting information




**Supporting File**: anie72945‐sup‐0001‐SuppMat.pdf.
The authors have cited additional references within the Supporting Information [63, 64, 65].

## Data Availability

The data that supports the findings of this study are available in the Supporting Information of this article.
