# Supplementary material for: On the Ability of Bismuth to Couple Weakly Coordinating Anions
Source: Angew Chem Int Ed Engl. 2026 May 30;65(29):e6127002. doi: 10.1002/anie.6127002 (PMC13360579; doi:10.1002/anie.6127002)
Supplement: Supplementary file 1 — Supporting File: anie72945‐sup‐0001‐SuppMat.pdf. The authors have cited additional references within the Supporting Information [63, 64, 65]. [file ANIE-65-e6127002-s001.pdf]

Supporting Information

On the Ability of Bismuth to Couple Weakly Coordinating Anions

Lucas Mele,[‡] Reena Balhara,[‡] Dimitrios A. Pantazis*and Josep Cornella*

Max-Planck-Institut für Kohlenforschung, Kaiser-Wilhelm-Platz 1, Mülheim an der Ruhr, 45470, Germany.

cornella@kofo.mpg.de

Table of contents

1. Computational Details 2
2. Benchmarking for relativistic computation 3
   2.1. Comparison of electronic energies 3
   2.2. Spin-Orbit Coupling 4
   2.3. Comparison with structures obtained by SC-XRD 4
3. Calculation on Bi^{3+} and Bi^{5+} ions 5
4. Calculation on the coupling of WCAs 5
5. Cartesian coordinates of the optimized geometries 9
6. References 42

1. Computational Details

All calculations were performed with ORCA 6.1.[42] All selected models were fully optimized for the present study at a common level of theory, using the B3LYP functional[32,33] and def2-TZVP(-f) basis sets[34] on all atoms. In these initial optimizations the ECP60MDF small-core effective core potential of Metz et al. was used for Bi[35] in combination with the valence basis set by Weigend et al.[34] The CPCM solvation model was used in optimizations with chloroform as solvent.[63] The resolution of the identity approximation for the Coulomb integrals with the def2/J auxiliary basis sets[64] and the chain-of-spheres approximation to exact exchange (RIJCOSX)[65] were used in all calculations. Relativity in these calculations is included indirectly (i.e., not explicitly in the electronic Hamiltonian) through the relativistic ECP for Bi. Tight SCF convergence settings were used. After optimization, single-point calculations were performed with three different all-electron scalar relativistic Hamiltonians: ZORA,[54,55] DKH2,[56,57] and X2C.[58,59] The calculations were performed with all-electron basis sets, including the all-electron SARC basis sets for Bi,[40] i.e. SARC-ZORA-TZVP for the ZORA Hamiltonian and SARC-DKH-TZVP for DKH2, and the x2c-TZVPAll[61] or SARC-DKH-TZVP basis set for the X2C Hamiltonian. Appropriate recontracted ZORA/DKH-def2-TZVP basis sets[39–41] or x2c-TZVPAll basis sets[61] were used for other atoms. The SARC/J or x2c/J auxiliary basis sets were employed in all-electron calculations.

The use of SARC basis sets, particularly for Bi,[40] proved to be a crucial technical detail because this basis set is flexible enough to enable SCF convergence in both relativistic and non-relativistic calculations (attempts with other relativistically adapted all-electron basis sets such as the x2c-TZVPall failed in converging to the non-relativistic limit). For this reason, all non-relativistic calculations were performed with the SARC-ZORA-TZVP basis sets. We note that the SARC-DKH basis sets are ideal for use with both the DKH2 and the X2C Hamiltonians, and are strongly recommended for the latter over the smaller and more heavily contracted x2c-TZVPall alternatives, particularly for accurate energetics and spectroscopic properties. To quantify geometric effects, full geometry optimizations were also performed with scalar relativistic Hamiltonians and with non-relativistic calculations. Intermediate scaling of the effects of relativity was investigated by adjusting the speed of light in multiples, thus effectively scaling the fine structure constant $a = 1/c$ from one to zero.

2. Benchmarking for relativistic computation

2.1. Comparison of electronic energies

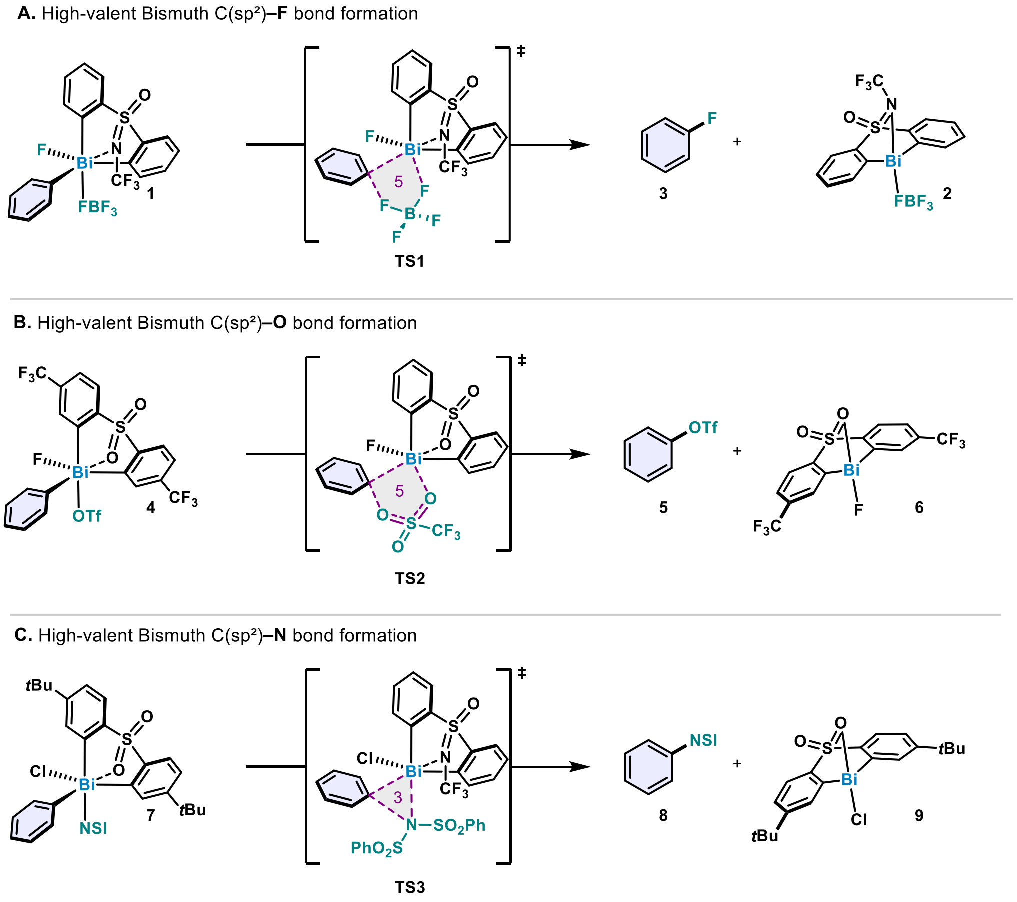

Figure S1 WCA coupling by hypervalent bismuth species.

Electronic energies of the reaction intermediates and transition states of Figure **S1** were calculated using various relativistic method and compared to non-relativistic values.

Table S1. Calculated electronic energies (kcal/mol) with the B3LYP functional and various treatments of relativistic effects, compared to non-relativistic values, for intermediates involved in the three reactions studied.

**C-F**	**1**	**TS1**	**2 + 3**
ECP	0.0	22.8	-40.1
ZORA	0.0	22.5	-44.8
DKH2	0.0	22.6	-44.5
X2C	0.0	22.7	-44.3
Non-relativistic	0.0	43.6	-12.2
**C-O**	**4**	**TS2**	**5 + 6**
ECP	0.0	19.7	-37.8
ZORA	0.0	18.4	-40.9
DKH2	0.0	18.5	-41.1
X2C	0.0	18.6	-40.4
Non-relativistic	0.0	39.1	-12.4
**C-N**	**7**	**TS3**	**8 + 9**
ECP	0.0	18.1	-61.9
ZORA	0.0	20.5	-60.4

DKH2	0.0	20.9	-60.7
X2C	0.0	20.9	-60.0
Non-relativistic	0.0	39.4	-34.2

The different relativistic treatments lead to quantitatively similar results. For the remaining of the study, ZORA approximation was favored due to it lower computation cost.

2.2. Spin-Orbit Coupling

Impact of Spin-Orbit Coupling (SOC) on the overall themodynamics was evaluated on the fluorination reaction by explicit inclusion of SOC in two-component X2C calculations conducted with Turbomole and appropriate x2c-TZVPall-2c basis sets.

Table S2. Calculated electronic energies (kcal/mol) with the B3LYP functional and various treatments of relativistic effects.

**C-F**	**1**	**2 + 3**
Scalar Relativistic	0.0	-44.3
Fully Relativistic	0.0	-45.5

2.3. Comparison with structures obtained by SC-XRD

Geometries obtained by ZORA calculation were compared with the experimentally observed one obtained by SC-XRD. Bi(III) product **2** and Bi(V) **10**, analog to **1**, were selected.

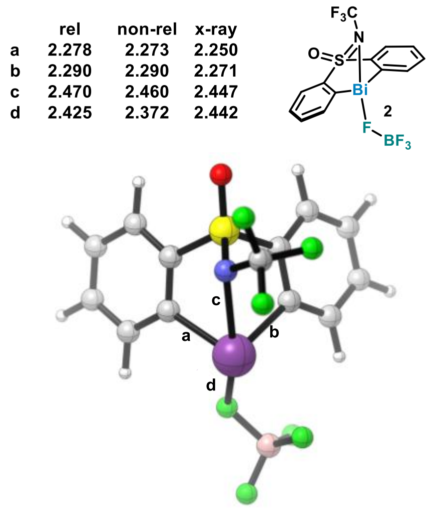

Figure S2 Experimental vs computed bond lengths (in Å) of **2**.

	rel	non-rel	x-ray
a	2.240	2.236	2.222
b	2.259	2.253	2.224
c	2.817	2.745	2.836
d	2.223	2.207	2.207
e	2.422	2.273	2.661
f	2.043	2.012	2.042
a'	2.240	2.236	
b'	2.259	2.253	
c'	2.815	2.746	
d'	2.223	2.207	
e'	2.419	2.273	
f'	2.043	2.012	

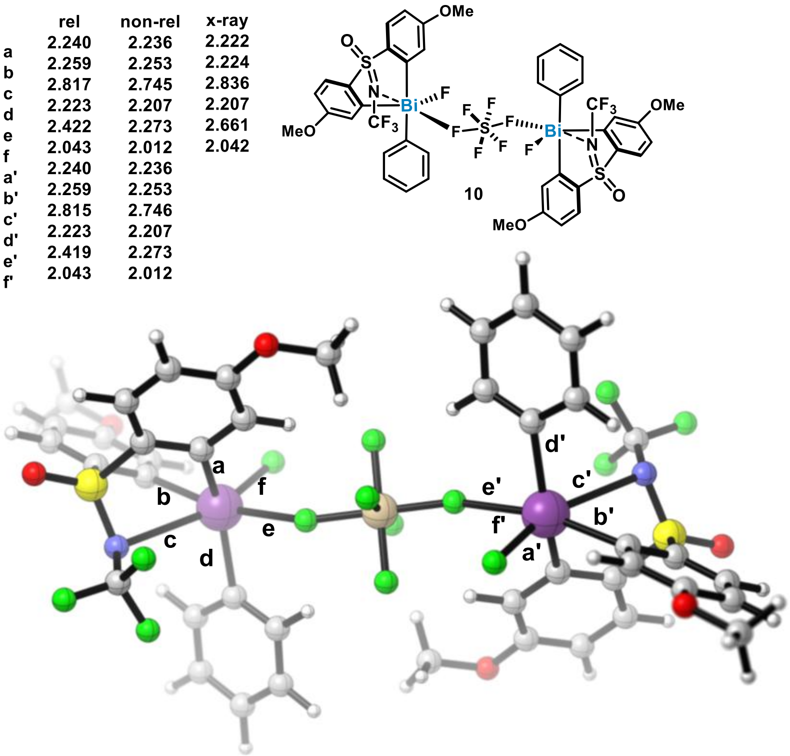

Figure S3 Experimental vs computed bond lengths (in Å) of **10**.

3. Calculation on Bi^{3+} and Bi^{5+} ions

Table S3. Radial Expectation Values (in Bohr) for Bi(III) and Bi(V) ions, obtained from ZORA and non-relativistic Hartree–Fock calculations with the SARC-ZORA-TZVP basis set

		**Bi(III)**	**Bi(V)**
5d	ZORA	1.209947	1.185696
	Non-relativistic	1.191921	1.175225
6s	ZORA	2.056286	2.000967
	Non-relativistic	2.289219	2.217076
6p	ZORA	2.675916	2.423757
	Non-relativistic	2.754178	2.510343

4. Calculation on the coupling of WCAs

Table S4. Relative energies (kcal/mol) of optimized geometries using the ZORA Hamiltonian with the B3LYP functional and all-electron SARC-ZORA-TZVP (Bi) and ZORA-def2-TZVP (other atoms) basis sets, with varying relativistic contribution, for the reductive elimination of fluorobenzene from fluorobismuthonium species **1**

**C-F**	**1**	**TS2**	**2 + 3**
Present study:	0.0	22.8 (24.4)	-40.1 (-41.4)
ZORA (normal)	0.0	21.8 (23.1)	-41.2 (-42.7)
ZORA with c/0.50	0.0	40.4 (42.0)	-15.2 (-18.0)
Non-relativistic	0.0	46.1 (47.1)	-7.3 (-9.9)

Values are given as A (B), where A and B are the relative electronic and free energies, respectively.

Table S5. Second-order perturbation stabilization energy (kcal/mol) from NBO analysis for the Bi(V) complex, **1** and Bi(III) complex, **2**

donor	acceptor	$E(2)_{rel}$		$E(2)_{non-rel}$	
		Bi(V)	Bi(III)	Bi(V)	Bi(III)
N2	Bi	9.63	12.65	11.16	12.18
N2	Bi	26.69	59.77	32.95	59.25
F1	Bi	1.82	4.61	3.97	4.62
F1	Bi	5.08	34.24	11.43	37.59
F1	Bi	0.07	2.64	0.08	2.14

Table S6. Calculated orbital energies (eV) for the valence s and p orbitals for the reductive elimination of fluorobenzene from fluorobismuthonium species, **1**

C-F	**1**		**2 + 3**	
Bi	E_{rel}	$E_{non-rel}$	E_{rel}	$E_{non-rel}$
6s	-13.5	-8.8	-14.7	-10.7
6px	-0.6	-0.3	-3.3	-3.0
6py	-1.6	-1.2	-2.8	-2.7
6pz	-1.8	-1.6	-3.0	-3.0
6p	-4.1	-3.1	-9.1	-8.7

*ZORA optimized geometries.

Table S7. Scaled values of electronic energies (kcal/mol) with adjusted speed of light using the B3LYP functional and the ZORA Hamiltonian with all-electron SARC-ZORA-TZVP and ZORA-def2-TZVP basis sets, with varying relativistic contribution

**C-F**	**1**	**TS1**	**2 + 3**
ZORA (normal)	0.0	22.5	-44.8
ZORA with c/0.75	0.0	33.0	-29.5
ZORA with c/0.50	0.0	39.3	-19.5
ZORA with c/0.25	0.0	42.6	-13.9
Non-relativistic	0.0	43.6	-12.2

**C-O**	**4**	**TS2**	**5 + 6**
ZORA (normal)	0.0	18.4	-40.9
ZORA with c/0.75	0.0	28.9	-27.6
ZORA with c/0.50	0.0	35.1	-18.9
ZORA with c/0.25	0.0	38.2	-13.9
Non-relativistic	0.0	39.1	-12.4

**C-N**	**7**	**TS3**	**8 + 9**
ZORA (normal)	0.0	20.5	-60.4

ZORA with c/0.75	0.0	30.4	-48.1
ZORA with c/0.50	0.0	35.9	-40.0
ZORA with c/0.25	0.0	38.7	-35.6
Non-relativistic	0.0	39.4	-34.2

Table S8. Orbital energies (a.u.) for the Bi-centered 6s orbitals for Bi(III) complexes at the B3LYP functional and the ZORA Hamiltonian with all-electron SARC-ZORA-TZVP and ZORA-def2-TZVP basis sets

**Bi(III)**	**E_{rel}**	**$E_{non\text{-}rel}$**	**$\Delta_{Enonrel\text{-}Erel}$**
**C-F (2)**	-0.61472	-0.52559	0.08913
**C-O (6)**	-0.56915	-0.51496	0.05419
**C-N (9)**	-0.59456	-0.48915	0.10541

Table S9. Orbital energies (a.u.) for HOMO and low-lying 6s acceptor orbital for the Bi(V) complexes at the B3LYP functional and the ZORA Hamiltonian with all-electron SARC-ZORA-TZVP and ZORA-def2-TZVP basis sets

**Bi(V)**	**E_{rel}**		**$E_{non\text{-}rel}$**	
	HOMO	LUMO	HOMO	LUMO +1
**C-F (1)**	-0.28741	-0.12917	-0.28068	-0.0334
**C-O (4)**	-0.27308	-0.13792	-0.28041	-0.09269
**C-N (7)**	-0.25389	-0.11074	-0.25758	-0.06596

Table S10. Calculated orbital energies (eV) for the valence s and p orbitals for the reductive elimination of fluorobenzene from fluorobismuthonium species, **1** at the B3LYP functional and the ZORA Hamiltonian with all-electron SARC-ZORA-TZVP and ZORA-def2-TZVP basis sets

C-F	**1**		**TS1**		**2 + 3**	
Bi	E_{rel}	$E_{non\text{-}rel}$	E_{rel}	$E_{non\text{-}rel}$	E_{rel}	$E_{non\text{-}rel}$
6s	-13.4	-9.1	-13.8	-9.9	-14.7	-10.7
6px	-0.6	-0.5	-2.0	-1.9	-3.3	-3.1
6py	-1.6	-1.6	-1.8	-1.9	-2.8	-2.6
6pz	-1.8	-2.8	-1.9	-2.1	-3.0	-2.9
6p	-1.3	-1.6	-1.9	-2.0	-3.0	-2.9

*single points on the default ECP optimized geometries

Table S11. Calculated (RIJCOSX-B3LYP/ZORA-def2-TZVP/SARC-ZORA-TZVP) orbital energies (eV) for the valence s and p orbitals for the C(sp^2) triflation at the B3LYP functional and the ZORA Hamiltonian with all-electron SARC-ZORA-TZVP and ZORA-def2-TZVP basis sets

C-O	**4**		**TS2**		**5 + 6**	
Bi	E_{rel}	$E_{non\text{-}rel}$	E_{rel}	$E_{non\text{-}rel}$	E_{rel}	$E_{non\text{-}rel}$
6s	-13.7	-9.3	-14.1	-10.2	-14.2	-10.3
6px	-1.9	-1.9	-2.1	-1.9	-2.6	-2.6
6py	-0.5	-0.4	-1.9	-1.8	-2.8	-2.5
6pz	-1.9	-1.9	-2.1	-2.0	-2.6	-2.6
6p	-1.4	-1.4	-2.0	-1.9	-2.7	-2.6

*single points on the default ECP optimized geometries

Table S12. Calculated (RIJCOSX-B3LYP/ZORA-def2-TZVP/SARC-ZORA-TZVP) orbital energies (eV) for the valence s and p orbitals for the ligand controlled chemodivergent reaction at the B3LYP functional and the ZORA Hamiltonian with all-electron SARC-ZORA-TZVP and ZORA-def2-TZVP basis sets

C-N	**7**		**TS3**		**8 + 9**	
Bi	E_{rel}	$E_{non-rel}$	E_{rel}	$E_{non-rel}$	E_{rel}	$E_{non-rel}$
6s	-12.9	-8.6	-13.5	-9.7	-13.9	-10.0
6px	-0.8	-0.7	-1.4	-1.6	-2.6	-2.5
6py	-0.8	-0.7	-0.8	-0.9	-2.8	-2.5
6pz	-1.8	-1.8	-2.2	-2.4	-2.6	-2.6
6p	-1.1	-1.1	-1.5	-1.6	-2.7	-2.5

*single points on the default ECP optimized geometries

5. Cartesian coordinates of the optimized geometries

1-ECP

Bi	1.990195	10.441765	14.084462
S	2.562165	7.271784	14.632735
F	2.947673	12.168176	14.612418
O	2.837051	5.867731	14.833065
F	-0.420193	7.889702	12.520090
C	2.321855	9.548881	16.112480
N	1.182414	7.777837	14.072613
F	1.484994	7.057470	11.878370
C	2.362755	10.274918	17.290169
H	2.141133	11.334019	17.291045
C	-0.102355	11.156410	13.934936
F	0.188401	5.914253	13.181129
C	3.824984	7.997571	13.599414
C	2.987466	7.536634	17.313211
H	3.225900	6.482169	17.302236
C	-2.685278	12.013988	13.922427
H	-3.712718	12.353564	13.912295
C	2.701364	9.629464	18.481182
C	3.013626	8.275185	18.493030
H	3.278622	7.786612	19.420894
C	3.688686	9.318870	13.177751
C	-1.705099	12.765767	13.283505
H	-1.962243	13.686843	12.777185
C	4.639306	9.914287	12.377750
H	4.533873	10.938247	12.052829
C	4.921725	7.243751	13.211444
H	5.012573	6.217095	13.537820
C	2.629411	8.191824	16.145948
C	-0.378778	12.339798	13.278963

H	0.385710	12.909172	12.774123
C	5.877930	7.831284	12.388164
H	6.732207	7.250776	12.067339
C	0.636019	7.171272	12.933179
C	-2.358322	10.828914	14.572698
H	-3.121966	10.243599	15.067834
C	-1.040258	10.379386	14.590672
H	-0.779471	9.456923	15.090458
C	5.738554	9.150186	11.977596
F	2.307899	11.854512	11.873157
H	2.725696	10.195118	19.403568
H	6.484472	9.600581	11.336228
B	1.750493	11.172959	10.715941
F	0.751530	11.967445	10.173978
F	1.220877	9.961720	11.210381
F	2.772085	10.933293	9.808718

TS1-ECP

Bi	1.879352	10.172863	14.037605
S	2.826918	7.067221	14.696621
F	2.797430	11.981100	14.508354
O	3.213161	5.694419	14.946702
F	-0.224165	7.364305	12.591235
C	2.375283	9.359336	16.102155
N	1.404367	7.421354	14.115091
F	1.778832	6.931260	11.869011
C	2.377266	10.122193	17.259878
H	2.071518	11.160263	17.231529
C	-0.581392	11.297072	13.228569
F	0.732493	5.477116	13.087624
C	4.034517	7.876431	13.661953

C	3.225878	7.451631	17.364423
H	3.554648	6.421611	17.381656
C	-2.611387	12.649933	14.156330
H	-3.450234	13.211200	14.544514
C	2.792150	9.553032	18.465111
C	3.213134	8.228964	18.518547
H	3.534652	7.796991	19.456733
C	3.768028	9.180285	13.254482
C	-1.482488	13.326607	13.711354
H	-1.419536	14.406431	13.751442
C	4.687873	9.830801	12.448974
H	4.503027	10.841246	12.110631
C	5.192935	7.205400	13.300068
H	5.366889	6.192878	13.636959
C	2.792472	8.035608	16.183675
C	-0.380270	12.629288	13.182991
H	0.508160	13.120143	12.817546
C	6.106714	7.866922	12.485758
H	7.012446	7.361434	12.178507
C	0.954890	6.808827	12.948167
C	-2.684176	11.262633	14.096074
H	-3.562996	10.728877	14.434219
C	-1.618159	10.507579	13.578143
H	-1.640393	9.432197	13.494510
C	5.855179	9.168020	12.065372
F	2.138141	11.620458	11.482255
H	2.790538	10.152981	19.366278
H	6.569326	9.675892	11.429875
B	1.146550	11.092690	10.626993
F	0.435373	12.109548	10.017593
F	0.230611	10.341653	11.497186
F	1.687715	10.200292	9.724778

2-ECP

Bi	1.393722	10.286029	14.176462
S	2.552832	7.375981	14.780061
O	2.850216	5.972310	14.939657
F	-0.457850	7.890089	12.631212
C	2.100233	9.601216	16.227084
N	1.119805	7.834087	14.210335
F	1.474247	7.099422	12.024896
C	2.135324	10.359878	17.387803
H	1.799583	11.388652	17.381430
F	0.195203	5.934942	13.324372
C	3.736866	8.218289	13.756453
C	3.048529	7.702185	17.448096
H	3.395538	6.677869	17.448231
C	2.618468	9.794630	18.569429
C	3.069356	8.478789	18.602580
H	3.438978	8.053260	19.525757
C	3.398210	9.517276	13.390473
C	4.301733	10.231181	12.617041
H	4.083346	11.243949	12.307970
C	4.926773	7.604793	13.399446
H	5.144258	6.592138	13.710320
C	2.552923	8.289643	16.295067
C	5.816689	8.334663	12.617693
H	6.750777	7.884939	12.308951
C	0.609209	7.193116	13.070101
C	5.504745	9.634723	12.233513
F	2.495086	12.336049	14.858848
H	2.644307	10.391514	19.472150
H	6.203709	10.195724	11.626449
B	2.618402	13.347737	13.784269

F	1.872312	14.446797	14.147485
F	2.087111	12.712764	12.642977
F	3.958133	13.631550	13.623798

3-ECP

C	9.359658	1.588294	2.851975
C	8.202827	1.233240	3.539508
C	10.512307	1.941968	3.546960
H	7.295042	0.955281	3.021125
H	11.408160	2.216893	3.005948
C	8.235417	1.243070	4.921954
C	10.511390	1.941708	4.938569
H	11.405394	2.216202	5.483468
C	9.363731	1.589474	5.642568
H	9.338507	1.582165	6.723934
H	9.355685	1.587335	1.769629
F	7.109622	0.898220	5.602607

1-zora

Bi	1.990195	10.441765	14.084462
S	2.562165	7.271784	14.632735
F	2.947673	12.168176	14.612418
O	2.837051	5.867731	14.833065
F	-0.420193	7.889702	12.520090
C	2.321855	9.548881	16.112480
N	1.182414	7.777837	14.072613
F	1.484994	7.057470	11.878370
C	2.362755	10.274918	17.290169
H	2.141133	11.334019	17.291045
C	-0.102355	11.156410	13.934936

F	0.188401	5.914253	13.181129
C	3.824984	7.997571	13.599414
C	2.987466	7.536634	17.313211
H	3.225900	6.482169	17.302236
C	-2.685278	12.013988	13.922427
H	-3.712718	12.353564	13.912295
C	2.701364	9.629464	18.481182
C	3.013626	8.275185	18.493030
H	3.278622	7.786612	19.420894
C	3.688686	9.318870	13.177751
C	-1.705099	12.765767	13.283505
H	-1.962243	13.686843	12.777185
C	4.639306	9.914287	12.377750
H	4.533873	10.938247	12.052829
C	4.921725	7.243751	13.211444
H	5.012573	6.217095	13.537820
C	2.629411	8.191824	16.145948
C	-0.378778	12.339798	13.278963
H	0.385710	12.909172	12.774123
C	5.877930	7.831284	12.388164
H	6.732207	7.250776	12.067339
C	0.636019	7.171272	12.933179
C	-2.358322	10.828914	14.572698
H	-3.121966	10.243599	15.067834
C	-1.040258	10.379386	14.590672
H	-0.779471	9.456923	15.090458
C	5.738554	9.150186	11.977596
F	2.307899	11.854512	11.873157
H	2.725696	10.195118	19.403568
H	6.484472	9.600581	11.336228
B	1.750493	11.172959	10.715941
F	0.751530	11.967445	10.173978
F	1.220877	9.961720	11.210381

F 2.772085 10.933293 9.808718

TS1-zora

Bi	1.879352	10.172863	14.037605
S	2.826918	7.067221	14.696621
F	2.797430	11.981100	14.508354
O	3.213161	5.694419	14.946702
F	-0.224165	7.364305	12.591235
C	2.375283	9.359336	16.102155
N	1.404367	7.421354	14.115091
F	1.778832	6.931260	11.869011
C	2.377266	10.122193	17.259878
H	2.071518	11.160263	17.231529
C	-0.581392	11.297072	13.228569
F	0.732493	5.477116	13.087624
C	4.034517	7.876431	13.661953
C	3.225878	7.451631	17.364423
H	3.554648	6.421611	17.381656
C	-2.611387	12.649933	14.156330
H	-3.450234	13.211200	14.544514
C	2.792150	9.553032	18.465111
C	3.213134	8.228964	18.518547
H	3.534652	7.796991	19.456733
C	3.768028	9.180285	13.254482
C	-1.482488	13.326607	13.711354
H	-1.419536	14.406431	13.751442
C	4.687873	9.830801	12.448974
H	4.503027	10.841246	12.110631
C	5.192935	7.205400	13.300068
H	5.366889	6.192878	13.636959
C	2.792472	8.035608	16.183675

C	-0.380270	12.629288	13.182991
H	0.508160	13.120143	12.817546
C	6.106714	7.866922	12.485758
H	7.012446	7.361434	12.178507
C	0.954890	6.808827	12.948167
C	-2.684176	11.262633	14.096074
H	-3.562996	10.728877	14.434219
C	-1.618159	10.507579	13.578143
H	-1.640393	9.432197	13.494510
C	5.855179	9.168020	12.065372
F	2.138141	11.620458	11.482255
H	2.790538	10.152981	19.366278
H	6.569326	9.675892	11.429875
B	1.146550	11.092690	10.626993
F	0.435373	12.109548	10.017593
F	0.230611	10.341653	11.497186
F	1.687715	10.200292	9.724778

2-zora

Bi	1.393722	10.286029	14.176462
S	2.552832	7.375981	14.780061
O	2.850216	5.972310	14.939657
F	-0.457850	7.890089	12.631212
C	2.100233	9.601216	16.227084
N	1.119805	7.834087	14.210335
F	1.474247	7.099422	12.024896
C	2.135324	10.359878	17.387803
H	1.799583	11.388652	17.381430
F	0.195203	5.934942	13.324372
C	3.736866	8.218289	13.756453
C	3.048529	7.702185	17.448096

H	3.395538	6.677869	17.448231
C	2.618468	9.794630	18.569429
C	3.069356	8.478789	18.602580
H	3.438978	8.053260	19.525757
C	3.398210	9.517276	13.390473
C	4.301733	10.231181	12.617041
H	4.083346	11.243949	12.307970
C	4.926773	7.604793	13.399446
H	5.144258	6.592138	13.710320
C	2.552923	8.289643	16.295067
C	5.816689	8.334663	12.617693
H	6.750777	7.884939	12.308951
C	0.609209	7.193116	13.070101
C	5.504745	9.634723	12.233513
F	2.495086	12.336049	14.858848
H	2.644307	10.391514	19.472150
H	6.203709	10.195724	11.626449
B	2.618402	13.347737	13.784269
F	1.872312	14.446797	14.147485
F	2.087111	12.712764	12.642977
F	3.958133	13.631550	13.623798

3-zora

C	9.359658	1.588294	2.851975
C	8.202827	1.233240	3.539508
C	10.512307	1.941968	3.546960
H	7.295042	0.955281	3.021125
H	11.408160	2.216893	3.005948
C	8.235417	1.243070	4.921954
C	10.511390	1.941708	4.938569
H	11.405394	2.216202	5.483468

C	9.363731	1.589474	5.642568
H	9.338507	1.582165	6.723934
H	9.355685	1.587335	1.769629
F	7.109622	0.898220	5.602607

1-zora-c/0.5

Bi	1.990195	10.441765	14.084462
S	2.562165	7.271784	14.632735
F	2.947673	12.168176	14.612418
O	2.837051	5.867731	14.833065
F	-0.420193	7.889702	12.520090
C	2.321855	9.548881	16.112480
N	1.182414	7.777837	14.072613
F	1.484994	7.057470	11.878370
C	2.362755	10.274918	17.290169
H	2.141133	11.334019	17.291045
C	-0.102355	11.156410	13.934936
F	0.188401	5.914253	13.181129
C	3.824984	7.997571	13.599414
C	2.987466	7.536634	17.313211
H	3.225900	6.482169	17.302236
C	-2.685278	12.013988	13.922427
H	-3.712718	12.353564	13.912295
C	2.701364	9.629464	18.481182
C	3.013626	8.275185	18.493030
H	3.278622	7.786612	19.420894
C	3.688686	9.318870	13.177751
C	-1.705099	12.765767	13.283505
H	-1.962243	13.686843	12.777185
C	4.639306	9.914287	12.377750
H	4.533873	10.938247	12.052829

C	4.921725	7.243751	13.211444
H	5.012573	6.217095	13.537820
C	2.629411	8.191824	16.145948
C	-0.378778	12.339798	13.278963
H	0.385710	12.909172	12.774123
C	5.877930	7.831284	12.388164
H	6.732207	7.250776	12.067339
C	0.636019	7.171272	12.933179
C	-2.358322	10.828914	14.572698
H	-3.121966	10.243599	15.067834
C	-1.040258	10.379386	14.590672
H	-0.779471	9.456923	15.090458
C	5.738554	9.150186	11.977596
F	2.307899	11.854512	11.873157
H	2.725696	10.195118	19.403568
H	6.484472	9.600581	11.336228
B	1.750493	11.172959	10.715941
F	0.751530	11.967445	10.173978
F	1.220877	9.961720	11.210381
F	2.772085	10.933293	9.808718

TS1-zora-c/0.5

Bi	1.879352	10.172863	14.037605
S	2.826918	7.067221	14.696621
F	2.797430	11.981100	14.508354
O	3.213161	5.694419	14.946702
F	-0.224165	7.364305	12.591235
C	2.375283	9.359336	16.102155
N	1.404367	7.421354	14.115091
F	1.778832	6.931260	11.869011
C	2.377266	10.122193	17.259878

H	2.071518	11.160263	17.231529
C	-0.581392	11.297072	13.228569
F	0.732493	5.477116	13.087624
C	4.034517	7.876431	13.661953
C	3.225878	7.451631	17.364423
H	3.554648	6.421611	17.381656
C	-2.611387	12.649933	14.156330
H	-3.450234	13.211200	14.544514
C	2.792150	9.553032	18.465111
C	3.213134	8.228964	18.518547
H	3.534652	7.796991	19.456733
C	3.768028	9.180285	13.254482
C	-1.482488	13.326607	13.711354
H	-1.419536	14.406431	13.751442
C	4.687873	9.830801	12.448974
H	4.503027	10.841246	12.110631
C	5.192935	7.205400	13.300068
H	5.366889	6.192878	13.636959
C	2.792472	8.035608	16.183675
C	-0.380270	12.629288	13.182991
H	0.508160	13.120143	12.817546
C	6.106714	7.866922	12.485758
H	7.012446	7.361434	12.178507
C	0.954890	6.808827	12.948167
C	-2.684176	11.262633	14.096074
H	-3.562996	10.728877	14.434219
C	-1.618159	10.507579	13.578143
H	-1.640393	9.432197	13.494510
C	5.855179	9.168020	12.065372
F	2.138141	11.620458	11.482255
H	2.790538	10.152981	19.366278
H	6.569326	9.675892	11.429875
B	1.146550	11.092690	10.626993

F	0.435373	12.109548	10.017593
F	0.230611	10.341653	11.497186
F	1.687715	10.200292	9.724778

2-zora-c/0.5

Bi	1.393722	10.286029	14.176462
S	2.552832	7.375981	14.780061
O	2.850216	5.972310	14.939657
F	-0.457850	7.890089	12.631212
C	2.100233	9.601216	16.227084
N	1.119805	7.834087	14.210335
F	1.474247	7.099422	12.024896
C	2.135324	10.359878	17.387803
H	1.799583	11.388652	17.381430
F	0.195203	5.934942	13.324372
C	3.736866	8.218289	13.756453
C	3.048529	7.702185	17.448096
H	3.395538	6.677869	17.448231
C	2.618468	9.794630	18.569429
C	3.069356	8.478789	18.602580
H	3.438978	8.053260	19.525757
C	3.398210	9.517276	13.390473
C	4.301733	10.231181	12.617041
H	4.083346	11.243949	12.307970
C	4.926773	7.604793	13.399446
H	5.144258	6.592138	13.710320
C	2.552923	8.289643	16.295067
C	5.816689	8.334663	12.617693
H	6.750777	7.884939	12.308951
C	0.609209	7.193116	13.070101
C	5.504745	9.634723	12.233513

F	2.495086	12.336049	14.858848
H	2.644307	10.391514	19.472150
H	6.203709	10.195724	11.626449
B	2.618402	13.347737	13.784269
F	1.872312	14.446797	14.147485
F	2.087111	12.712764	12.642977
F	3.958133	13.631550	13.623798

3-zora-c/0.5

C	9.359658	1.588294	2.851975
C	8.202827	1.233240	3.539508
C	10.512307	1.941968	3.546960
H	7.295042	0.955281	3.021125
H	11.408160	2.216893	3.005948
C	8.235417	1.243070	4.921954
C	10.511390	1.941708	4.938569
H	11.405394	2.216202	5.483468
C	9.363731	1.589474	5.642568
H	9.338507	1.582165	6.723934
H	9.355685	1.587335	1.769629
F	7.109622	0.898220	5.602607

1-non-relativistic

Bi	1.990195	10.441765	14.084462
S	2.562165	7.271784	14.632735
F	2.947673	12.168176	14.612418
O	2.837051	5.867731	14.833065
F	-0.420193	7.889702	12.520090
C	2.321855	9.548881	16.112480

N	1.182414	7.777837	14.072613
F	1.484994	7.057470	11.878370
C	2.362755	10.274918	17.290169
H	2.141133	11.334019	17.291045
C	-0.102355	11.156410	13.934936
F	0.188401	5.914253	13.181129
C	3.824984	7.997571	13.599414
C	2.987466	7.536634	17.313211
H	3.225900	6.482169	17.302236
C	-2.685278	12.013988	13.922427
H	-3.712718	12.353564	13.912295
C	2.701364	9.629464	18.481182
C	3.013626	8.275185	18.493030
H	3.278622	7.786612	19.420894
C	3.688686	9.318870	13.177751
C	-1.705099	12.765767	13.283505
H	-1.962243	13.686843	12.777185
C	4.639306	9.914287	12.377750
H	4.533873	10.938247	12.052829
C	4.921725	7.243751	13.211444
H	5.012573	6.217095	13.537820
C	2.629411	8.191824	16.145948
C	-0.378778	12.339798	13.278963
H	0.385710	12.909172	12.774123
C	5.877930	7.831284	12.388164
H	6.732207	7.250776	12.067339
C	0.636019	7.171272	12.933179
C	-2.358322	10.828914	14.572698
H	-3.121966	10.243599	15.067834
C	-1.040258	10.379386	14.590672
H	-0.779471	9.456923	15.090458
C	5.738554	9.150186	11.977596
F	2.307899	11.854512	11.873157

H	2.725696	10.195118	19.403568
H	6.484472	9.600581	11.336228
B	1.750493	11.172959	10.715941
F	0.751530	11.967445	10.173978
F	1.220877	9.961720	11.210381
F	2.772085	10.933293	9.808718

TS1-non-relativistic

Bi	1.879352	10.172863	14.037605
S	2.826918	7.067221	14.696621
F	2.797430	11.981100	14.508354
O	3.213161	5.694419	14.946702
F	-0.224165	7.364305	12.591235
C	2.375283	9.359336	16.102155
N	1.404367	7.421354	14.115091
F	1.778832	6.931260	11.869011
C	2.377266	10.122193	17.259878
H	2.071518	11.160263	17.231529
C	-0.581392	11.297072	13.228569
F	0.732493	5.477116	13.087624
C	4.034517	7.876431	13.661953
C	3.225878	7.451631	17.364423
H	3.554648	6.421611	17.381656
C	-2.611387	12.649933	14.156330
H	-3.450234	13.211200	14.544514
C	2.792150	9.553032	18.465111
C	3.213134	8.228964	18.518547
H	3.534652	7.796991	19.456733
C	3.768028	9.180285	13.254482
C	-1.482488	13.326607	13.711354
H	-1.419536	14.406431	13.751442

C	4.687873	9.830801	12.448974
H	4.503027	10.841246	12.110631
C	5.192935	7.205400	13.300068
H	5.366889	6.192878	13.636959
C	2.792472	8.035608	16.183675
C	-0.380270	12.629288	13.182991
H	0.508160	13.120143	12.817546
C	6.106714	7.866922	12.485758
H	7.012446	7.361434	12.178507
C	0.954890	6.808827	12.948167
C	-2.684176	11.262633	14.096074
H	-3.562996	10.728877	14.434219
C	-1.618159	10.507579	13.578143
H	-1.640393	9.432197	13.494510
C	5.855179	9.168020	12.065372
F	2.138141	11.620458	11.482255
H	2.790538	10.152981	19.366278
H	6.569326	9.675892	11.429875
B	1.146550	11.092690	10.626993
F	0.435373	12.109548	10.017593
F	0.230611	10.341653	11.497186
F	1.687715	10.200292	9.724778

2-non-relativistic

Bi	1.393722	10.286029	14.176462
S	2.552832	7.375981	14.780061
O	2.850216	5.972310	14.939657
F	-0.457850	7.890089	12.631212
C	2.100233	9.601216	16.227084
N	1.119805	7.834087	14.210335
F	1.474247	7.099422	12.024896

C	2.135324	10.359878	17.387803
H	1.799583	11.388652	17.381430
F	0.195203	5.934942	13.324372
C	3.736866	8.218289	13.756453
C	3.048529	7.702185	17.448096
H	3.395538	6.677869	17.448231
C	2.618468	9.794630	18.569429
C	3.069356	8.478789	18.602580
H	3.438978	8.053260	19.525757
C	3.398210	9.517276	13.390473
C	4.301733	10.231181	12.617041
H	4.083346	11.243949	12.307970
C	4.926773	7.604793	13.399446
H	5.144258	6.592138	13.710320
C	2.552923	8.289643	16.295067
C	5.816689	8.334663	12.617693
H	6.750777	7.884939	12.308951
C	0.609209	7.193116	13.070101
C	5.504745	9.634723	12.233513
F	2.495086	12.336049	14.858848
H	2.644307	10.391514	19.472150
H	6.203709	10.195724	11.626449
B	2.618402	13.347737	13.784269
F	1.872312	14.446797	14.147485
F	2.087111	12.712764	12.642977
F	3.958133	13.631550	13.623798

3-non-relativistic

C	9.359658	1.588294	2.851975
C	8.202827	1.233240	3.539508
C	10.512307	1.941968	3.546960

H	7.295042	0.955281	3.021125
H	11.408160	2.216893	3.005948
C	8.235417	1.243070	4.921954
C	10.511390	1.941708	4.938569
H	11.405394	2.216202	5.483468
C	9.363731	1.589474	5.642568
H	9.338507	1.582165	6.723934
H	9.355685	1.587335	1.769629
F	7.109622	0.898220	5.602607

4

Bi	3.799167	4.297904	6.274114
S	6.743357	2.997250	6.101641
O	6.361179	4.037566	7.065744
C	5.128227	4.381017	4.439172
C	6.339191	3.694669	4.517226
C	7.190239	3.564512	3.433322
H	8.120840	3.021747	3.523276
C	6.821456	4.160222	2.231894
H	7.471252	4.081449	1.372231
C	5.616562	4.846595	2.140304
C	4.758671	4.955917	3.237939
H	3.815421	5.476385	3.140311
C	4.191082	2.119212	6.511761
C	5.511971	1.727374	6.320760
C	5.852702	0.383935	6.301613
H	6.882412	0.085335	6.162763
C	4.849636	-0.561452	6.476671
H	5.100330	-1.613258	6.473360
C	3.530339	-0.157052	6.648689
C	3.183000	1.193965	6.663795
H	2.158380	1.503504	6.803431

C	3.709008	6.337121	7.136258
C	4.904096	7.027732	7.209536
H	5.846482	6.585192	6.919098
C	4.847021	8.332730	7.693636
H	5.762050	8.904381	7.776299
C	3.628682	8.889721	8.067926
H	3.596611	9.904058	8.443485
C	2.451670	8.156102	7.966223
H	1.505044	8.591366	8.258100
C	2.476168	6.846232	7.492505
H	1.569877	6.263493	7.423528
O	8.066775	2.433811	6.110616
C	5.233282	5.520446	0.848158
F	5.568098	6.833051	0.859618
F	5.844972	4.969629	-0.218614
F	3.904662	5.460646	0.623079
C	2.469171	-1.207845	6.859036
F	1.225465	-0.718445	6.711922
F	2.605321	-2.231243	5.988331
F	2.545640	-1.739102	8.100424
S	2.495095	3.883381	9.403672
F	2.394398	2.267600	11.487251
F	0.738538	2.024088	10.100473
F	2.701102	1.237355	9.601827
O	1.950586	3.753162	8.027397
O	3.950796	3.911536	9.399426
O	1.813381	4.858929	10.220932
C	2.054226	2.252140	10.193141
F	2.106323	4.259080	5.141376

TS2

Bi	3.864973	4.166762	6.436235
S	6.726614	2.628576	6.138692
O	6.411777	3.579113	7.215102
C	5.244983	4.297690	4.611370
C	6.402085	3.526110	4.635375
C	7.266380	3.437568	3.554543
H	8.153592	2.821814	3.605699
C	6.967967	4.170265	2.413601
H	7.631691	4.132523	1.560877
C	5.815622	4.950516	2.373699
C	4.946374	5.013055	3.462686
H	4.047365	5.610820	3.402629
C	4.120452	1.907873	6.385237
C	5.412559	1.425665	6.204075
C	5.683826	0.070979	6.099305
H	6.697346	-0.278178	5.958885
C	4.625390	-0.824980	6.199373
H	4.813465	-1.887113	6.136819
C	3.332465	-0.350860	6.389636
C	3.068518	1.017626	6.479154
H	2.057983	1.369620	6.633631
C	3.422248	6.398610	7.848660
C	4.579922	7.072837	8.070334
H	5.515453	6.604034	8.335130
C	4.448361	8.458280	7.906692
H	5.328402	9.073556	8.043282
C	3.218494	9.023668	7.587664
H	3.135247	10.096320	7.477916
C	2.092976	8.226599	7.419729
H	1.131480	8.659699	7.175525
C	2.167103	6.833289	7.569530
H	1.307155	6.191839	7.451299
O	8.015153	1.983491	6.093264

C	5.492191	5.697108	1.106754
F	6.588271	6.275478	0.567960
F	4.988256	4.868624	0.159831
F	4.581897	6.671450	1.298090
C	2.181068	-1.319108	6.463367
F	1.227700	-0.906407	7.323362
F	1.580499	-1.465754	5.256964
F	2.569119	-2.549141	6.856587
S	2.293039	3.947910	9.591869
F	3.580663	2.712989	11.550303
F	2.335559	1.413435	10.331253
F	4.232502	2.139410	9.558838
O	1.833166	3.500973	8.276809
O	3.401823	4.931042	9.478305
O	1.270831	4.278996	10.550029
C	3.166846	2.460064	10.305789
F	2.195082	4.217527	5.217304

5

C	1.925280	6.004257	5.824803
C	1.370240	4.924083	6.485853
C	2.768155	6.909938	6.443139
C	1.684840	4.740569	7.828077
C	3.077460	6.709455	7.783985
C	2.539173	5.628069	8.474530
H	0.707138	4.249188	5.962776
H	3.170209	7.746888	5.890093
H	1.260558	3.902387	8.364819
H	3.737626	7.403658	8.286907
H	2.782172	5.479630	9.518502
O	1.535583	6.224360	4.474401
S	2.542519	5.947590	3.270335

O	3.904177	5.995937	3.702138
O	2.049223	6.705545	2.166355
C	2.158400	4.155692	2.893558
F	2.519699	3.384964	3.917850
F	0.856859	4.005648	2.664599
F	2.844118	3.799912	1.809612

6

Bi	5.972372	5.119051	4.206954
S	7.145185	2.202608	5.182605
O	7.718459	0.975891	5.682602
C	7.026173	3.589547	2.870115
C	7.437255	2.388037	3.435318
C	8.018494	1.365475	2.700408
H	8.315723	0.441582	3.176903
C	8.213686	1.564368	1.340480
H	8.672203	0.789168	0.742090
C	7.808092	2.760024	0.752987
C	7.213397	3.770838	1.507229
H	6.890356	4.682778	1.023872
C	4.715273	3.351711	4.937215
C	5.367512	2.174597	5.287411
C	4.697338	1.023277	5.670860
H	5.242461	0.126111	5.929497
C	3.309746	1.057913	5.723076
H	2.759243	0.180348	6.031486
C	2.636236	2.225511	5.376935
C	3.329232	3.369311	4.978512
H	2.781533	4.258914	4.697437
O	7.538220	3.492180	5.768833
C	8.053055	2.959916	-0.718349
F	9.355405	3.242247	-0.971412

F	7.754822	1.853328	-1.435377
F	7.328424	3.972533	-1.232486
C	1.131217	2.232952	5.380107
F	0.623035	3.467502	5.572098
F	0.630754	1.785275	4.201672
F	0.618270	1.437641	6.343303
F	4.576782	5.510502	2.707544

7

O	3.958763	-1.875452	-1.594250
S	2.740752	-1.390779	-0.977687
C	2.492589	0.292962	-1.507938
C	3.591156	0.962935	-2.026266
C	3.524155	2.333074	-2.253587
C	2.368714	3.061681	-1.976861
C	2.269862	4.575032	-2.160157
C	1.258270	2.359723	-1.488240
C	1.321292	1.002660	-1.245468
Bi	-0.555074	-0.068487	-0.747141
Cl	-1.135050	0.084146	-3.323360
C	0.055850	-2.140356	-1.293135
C	-0.934372	-3.060039	-1.585643
C	-0.609280	-4.294388	-2.151517
C	-1.667866	-5.355150	-2.451800
C	0.736156	-4.541865	-2.444651
C	1.725516	-3.615426	-2.163296
C	1.391670	-2.399250	-1.570886
O	2.628889	-1.393963	0.465728
H	4.502134	0.417718	-2.228158
H	4.400000	2.826909	-2.646395
H	0.343963	2.895614	-1.278171
H	-1.961828	-2.819183	-1.366082

H	1.026454	-5.479908	-2.896987
H	2.762341	-3.830607	-2.379638
O	-1.760731	-1.051334	3.449420
O	-0.005243	1.137335	3.781743
S	-0.937409	-1.470736	2.341661
C	4.377824	1.496014	1.599652
C	4.957531	0.579791	2.469022
C	0.971487	-3.403655	2.071276
C	2.998949	1.662862	1.581105
C	4.160922	-0.162462	3.337465
C	0.291269	-2.582474	2.962131
C	1.956362	-4.250711	2.557669
H	4.994392	2.080276	0.929512
H	6.031718	0.446062	2.475747
H	0.738099	-3.377076	1.018638
C	2.784347	0.001047	3.330395
C	2.215640	0.903873	2.439042
C	0.562936	-2.601508	4.325657
C	2.249928	-4.272079	3.918220
C	1.552087	-3.453354	4.800650
H	2.496497	-4.890634	1.872577
O	-1.599130	-2.104430	1.208949
S	0.453839	1.103274	2.413730
H	2.536276	2.368401	0.910255
H	4.611468	-0.869842	4.021276
H	0.011931	-1.957517	4.995824
H	3.021681	-4.932432	4.292361
H	2.164228	-0.568456	4.005397
H	1.776074	-3.475994	5.858965
N	-0.143892	-0.222615	1.618782
O	0.156194	2.217050	1.534899
C	-2.372383	1.168387	-0.305834
C	-2.692112	2.140965	-1.231255

C	-3.058993	1.011937	0.882816
C	-4.115582	1.875400	1.138272
C	-4.477193	2.890775	0.245245
C	-3.748038	3.002954	-0.937665
H	-3.984739	3.768635	-1.661333
H	-2.157175	2.239617	-2.164095
H	-2.809889	0.256549	1.608715
H	-4.658312	1.745374	2.064686
C	-5.635536	3.826664	0.592954
C	-5.314372	4.554571	1.912640
H	-4.400993	5.144507	1.814869
H	-5.179744	3.854273	2.737417
H	-6.132851	5.229337	2.171916
C	-6.922610	2.997225	0.761990
C	-5.877214	4.880299	-0.494452
H	-7.165193	2.468287	-0.161820
H	-7.757478	3.656415	1.008860
H	-6.826859	2.261040	1.560763
H	-6.707697	5.521638	-0.195418
H	-6.136885	4.421735	-1.450413
H	-5.001587	5.515065	-0.642257
C	3.551642	5.170248	-2.753291
C	2.019427	5.217188	-0.780715
C	1.096065	4.901495	-3.102979
C	-1.645443	-5.676884	-3.958359
C	-3.079097	-4.892176	-2.072678
C	-1.334653	-6.626194	-1.646168
H	-1.336473	-6.415851	-0.574947
H	-0.356301	-7.025918	-1.914799
H	-2.081862	-7.396516	-1.846835
H	-3.790092	-5.686167	-2.304767
H	-3.376282	-4.003699	-2.632981
H	-3.160117	-4.672435	-1.006494

H	-2.402129	-6.430795	-4.184412
H	-0.677008	-6.067030	-4.272973
H	-1.862612	-4.784133	-4.547734
H	3.426234	6.247663	-2.869253
H	3.772363	4.750380	-3.736462
H	4.413158	5.002989	-2.104305
H	1.244969	4.439765	-4.080862
H	1.025257	5.982199	-3.240202
H	0.144447	4.551485	-2.702352
H	1.096368	4.852722	-0.328516
H	1.941536	6.301110	-0.886122
H	2.841836	4.996015	-0.097714

TS3

O	-2.697878	-5.589226	1.155273
S	-1.935379	-4.521488	1.759363
C	-2.997926	-3.230978	2.352454
C	-4.375978	-3.315304	2.261442
C	-5.137262	-2.213050	2.645566
C	-4.541657	-1.046373	3.127891
C	-5.342528	0.193351	3.527569
C	-3.141866	-1.016601	3.239594
C	-2.369101	-2.093070	2.852840
Bi	-0.102081	-2.117983	2.972339
Cl	-0.022689	-0.998007	5.234183
C	-0.144229	-4.251850	3.787849
C	0.596231	-4.661092	4.879261
C	0.432760	-5.953479	5.396585
C	1.241310	-6.456676	6.593544
C	-0.503547	-6.795181	4.783716
C	-1.255315	-6.385240	3.692153

C	-1.058658	-5.106259	3.188571
O	-0.910572	-3.813980	0.974142
H	-4.848760	-4.209344	1.877639
H	-6.210795	-2.277493	2.551593
H	-2.656627	-0.130984	3.627921
H	1.296806	-3.976940	5.333639
H	-0.652116	-7.797818	5.159981
H	-1.972239	-7.048423	3.226930
O	2.781245	-1.551543	2.995206
O	0.575791	-1.520071	-1.321196
S	3.270727	-1.709457	1.628561
C	2.984965	2.382893	-1.399594
C	3.781187	2.339529	-2.540178
C	4.484428	0.700689	2.149637
C	2.431156	1.216914	-0.890821
C	4.023364	1.126111	-3.174784
C	4.447299	-0.415958	1.323753
C	5.366116	1.732371	1.854088
H	2.800281	3.323336	-0.897011
H	4.217847	3.250085	-2.930107
H	3.848680	0.754696	3.019669
C	3.478669	-0.047311	-2.668344
C	2.685563	0.008899	-1.528344
C	5.288330	-0.526387	0.220746
C	6.192828	1.645161	0.739028
C	6.156843	0.515435	-0.072238
H	5.406052	2.602902	2.495613
O	3.899143	-2.965297	1.294529
S	1.969315	-1.510314	-0.925573
H	1.833050	1.249913	0.004696
H	4.646612	1.088487	-4.058764
H	5.255782	-1.409142	-0.401129
H	6.870221	2.456365	0.505192

H	3.678654	-0.998822	-3.139798
H	6.802893	0.444271	-0.936969
N	1.955259	-1.410701	0.707282
O	2.807900	-2.589988	-1.400123
C	0.341713	-0.026898	1.618175
C	-0.657430	0.148997	0.707437
C	0.923121	0.929569	2.404431
C	0.412705	2.216475	2.251463
C	-0.616220	2.506965	1.347842
C	-1.140597	1.457468	0.595722
H	-1.942566	1.622146	-0.108445
H	-1.054136	-0.648995	0.101594
H	1.694571	0.707593	3.122436
H	0.842632	2.995298	2.866782
C	-1.117206	3.946061	1.212819
C	0.050040	4.836404	0.745238
H	0.433738	4.494284	-0.218037
H	0.873121	4.826764	1.460877
H	-0.289180	5.868381	0.634119
C	-1.627464	4.436646	2.581024
C	-2.261047	4.068952	0.199516
H	-2.452855	3.813371	2.930483
H	-1.985887	5.464584	2.496254
H	-0.841189	4.415243	3.336180
H	-2.583034	5.110062	0.146785
H	-3.123665	3.467065	0.491644
H	-1.949780	3.764731	-0.801506
C	-6.849043	0.007309	3.316192
C	-4.871203	1.388388	2.674494
C	-5.089974	0.497361	5.017069
C	0.273521	-6.865419	7.720507
C	2.198335	-5.390166	7.139323
C	2.071198	-7.679376	6.155935

H	2.762821	-7.408793	5.355929
H	1.436751	-8.491075	5.798598
H	2.652953	-8.051797	7.001577
H	2.742928	-5.800902	7.990623
H	1.661659	-4.503460	7.481653
H	2.930543	-5.083553	6.390257
H	0.842243	-7.225756	8.580058
H	-0.401505	-7.661657	7.405304
H	-0.329310	-6.013317	8.040591
H	-7.368982	0.919945	3.611527
H	-7.242700	-0.811530	3.921154
H	-7.088385	-0.190463	2.269656
H	-5.407194	-0.339772	5.641955
H	-5.656547	1.382110	5.314896
H	-4.035142	0.689499	5.215194
H	-3.812460	1.599967	2.826145
H	-5.435737	2.282495	2.946952
H	-5.029555	1.192808	1.612100

8

N	0.297137	-0.189393	1.132498
C	-0.803983	0.663772	0.770777
S	1.864495	0.414180	0.892753
S	-0.012757	-1.807077	1.598913
C	0.625972	-2.802674	0.284398
C	1.846639	-3.446945	0.444778
C	2.349227	-4.199198	-0.609406
C	1.635588	-4.298696	-1.799581
C	0.407598	-3.657899	-1.939911
C	-0.107616	-2.906482	-0.893875
H	-1.064689	-2.413975	-0.987241

H	2.392210	-3.346661	1.370336
H	-0.150191	-3.744215	-2.862849
H	3.300300	-4.703186	-0.500546
H	2.034973	-4.881454	-2.619542
C	1.965479	0.619590	-0.860308
C	2.340327	-0.464641	-1.647260
C	1.623896	1.845901	-1.420665
C	2.360140	-0.315495	-3.026728
C	2.012559	0.903195	-3.602689
C	1.651043	1.982358	-2.801970
H	1.337935	2.671321	-0.785422
H	2.649279	-1.151505	-3.649565
H	2.617363	-1.402776	-1.190975
H	2.028473	1.013621	-4.679350
H	1.387140	2.930417	-3.251489
O	1.903752	1.716356	1.503632
O	2.770718	-0.623494	1.306698
O	-1.450825	-1.888602	1.594296
O	0.725852	-2.086103	2.799063
C	-1.343110	1.517305	1.720044
C	-1.312612	0.651966	-0.523640
C	-2.361705	1.492276	-0.854984
C	-2.924276	2.366890	0.083402
C	-2.392525	2.360909	1.374219
H	-0.937946	1.520614	2.722713
H	-2.792962	3.016391	2.132927
H	-0.884581	-0.006722	-1.265808
H	-2.744342	1.466006	-1.866239
C	-4.081264	3.278202	-0.330535
C	-4.555573	4.175806	0.818362
C	-5.267664	2.408922	-0.789967
C	-3.626521	4.179928	-1.494091
H	-6.102507	3.046286	-1.089518

H	-5.605243	1.758857	0.019621
H	-5.000407	1.781060	-1.640727
H	-4.922891	3.590095	1.663008
H	-5.374278	4.806928	0.468676
H	-3.757535	4.830155	1.173945
H	-2.788131	4.809795	-1.189584
H	-4.448045	4.829608	-1.803567
H	-3.315273	3.594406	-2.359746

9

Cl	-0.000756	-1.214930	2.633849
Bi	-0.000217	-1.609155	0.106806
C	-1.559589	0.021418	-0.253153
C	-2.614552	0.286620	0.599882
C	-3.508488	1.343681	0.355536
C	-4.652795	1.595633	1.338511
C	-5.540787	0.338480	1.412573
C	-4.061619	1.889921	2.731234
C	-5.528106	2.783401	0.922751
C	-3.302616	2.131468	-0.778075
C	-2.240311	1.890587	-1.644985
C	-1.390809	0.832331	-1.373738
S	0.000488	0.473215	-2.410413
O	0.000412	-0.995002	-2.548649
O	0.000787	1.298263	-3.597837
C	1.559543	0.021216	-0.252475
C	1.391406	0.832125	-1.373160
C	2.241194	1.890238	-1.644052
C	3.303142	2.130976	-0.776665
C	3.508367	1.343201	0.357071
C	4.652132	1.595152	1.340678

C	2.614161	0.286279	0.601032
H	-2.743979	-0.323321	1.484179
H	-4.980261	-0.530908	1.757291
H	-5.963811	0.105723	0.433257
H	-6.363884	0.507384	2.110087
H	-4.868284	2.072736	3.444284
H	-3.423658	2.775409	2.700607
H	-3.466245	1.055090	3.101601
H	-4.955301	3.711577	0.879041
H	-6.324129	2.917149	1.656981
H	-5.995669	2.620060	-0.050105
H	-3.968229	2.951851	-0.999993
H	-2.082708	2.511366	-2.516760
H	2.084070	2.511026	-2.515906
H	3.968985	2.951245	-0.998308
C	5.539533	0.337668	1.416061
C	4.060068	1.890445	2.732816
C	5.528251	2.782310	0.924878
H	2.743068	-0.323638	1.485423
H	3.422479	2.776165	2.701223
H	4.866277	2.073332	3.446365
H	3.464084	1.056042	3.103172
H	5.963083	0.104137	0.437157
H	4.978434	-0.531275	1.760965
H	6.362259	0.506646	2.113993
H	6.323932	2.916011	1.659488
H	4.955927	3.710746	0.880435
H	5.996268	2.618308	-0.047647

6. References

[32] A. D. Becke, "Density-functional thermochemistry. III. The role of exact exchange" *J. Chem. Phys.* **1993**, *98*, 5648–5652.

[33] C. Lee, W. Yang, R. G. Parr, "Development of the Colle–Salvetti correlation-energy formula into a functional of the electron density" *Phys. Rev. B* **1988**, *37*, 785–789.

[34] F. Weigend, R. Ahlrichs, "Balanced basis sets of split valence, triple zeta valence and quadruple zeta valence quality for H to Rn: Design and assessment of accuracy" *Phys. Chem. Chem. Phys.* **2005**, *7*, 3297–3305.

[35] B. Metz, H. Stoll, M. Dolg, "Small-core multiconfiguration-Dirac–Hartree–Fock-adjusted pseudopotentials for post-d main group elements: Application to PbH and PbO" *J. Chem. Phys.* **2000**, *113*, 2563–2569.

[39] D. A. Pantazis, X. Y. Chen, C. R. Landis, F. Neese, "All-electron scalar relativistic basis sets for third-row transition metal atoms" *J. Chem. Theory Comput.* **2008**, *4*, 908–919.

[40] D. A. Pantazis, F. Neese, "All-electron scalar relativistic basis sets for the 6p elements" *Theor. Chem. Acc.* **2012**, *131*, 1292.

[41] J. D. Rolfes, F. Neese, D. A. Pantazis, "All-electron scalar relativistic basis sets for the elements Rb–Xe", *J. Comput. Chem.* **2020**, *41*, 1842–1849.

[42] F. Neese, "Software update: The ORCA program system—Version 6.0" *WIREs Comput. Mol. Sci.* **2025**, *15*, e70019.

[54] E. van Lenthe, E. J. Baerends, J. G. Snijders, "Relativistic regular two-component Hamiltonians" *J. Chem. Phys.* **1993**, *99*, 4597–4610.

[55] C. van Wüllen, "Molecular density functional calculations in the regular relativistic approximation: Method, application to coinage metal diatomics, hydrides, fluorides and chlorides, and comparison with first-order relativistic calculations" *J. Chem. Phys.* **1998**, *109*, 392–399.

[56] T. Nakajima, K. Hirao, "The Douglas–Kroll–Hess approach" *Chem. Rev.* **2012**, *112*, 385–402.

[57] B. A. Hess, "Relativistic electronic-structure calculations employing a two-component no-pair formalism with external-field projection operators" *Phys. Rev. A* **1986**, *33*, 3742–3748.

[58] M. Iliaš, T. Saue, "An infinite-order two-component relativistic Hamiltonian by a simple one-step transformation" *J. Chem. Phys.* **2007**, *126*, 064102.

[59] W. Liu, D. Peng, "Exact two-component Hamiltonians revisited" *J. Chem. Phys.* **2009**, *131*, 031104.

[61] P. Pollak, F. Weigend, "Segmented Contracted Error-Consistent Basis Sets of Double- and Triple-ζ Valence Quality for One- and Two-Component Relativistic All-Electron Calculations" *J. Chem. Theory Comput.* **2017**, *13*, 3696–3705.

[63] J. Tomasi, B. Mennucci, R. Cammi, "Quantum mechanical continuum solvation models" *Chem. Rev.* **2005**, *105*, 2999–3094.

[64] F. Weigend, "Accurate Coulomb-fitting basis sets for H to Rn" *Phys. Chem. Chem. Phys.* **2006**, *8*, 1057–1065.

[65] F. Neese, F. Wennmohs, A. Hansen, U. Becker, "Efficient, approximate and parallel Hartree–Fock and hybrid DFT calculations: A 'chain-of-spheres' algorithm for the Hartree–Fock exchange" *Chem. Phys.* **2009**, *356*, 98–109.
